# Supplementary material for: Tai chi mind-body exercise in patients with COPD: study protocol for a randomized controlled trial
Source: Trials. 2014 Aug 28;15:337. doi: 10.1186/1745-6215-15-337 (PMC4158042; doi:10.1186/1745-6215-15-337)
Supplement: Supplementary file 1 — Additional file 1: List of approving Institutional Review Boards.(DOCX 13 KB) [file 13063_2014_2212_MOESM1_ESM.docx]

**Additional file 1: List of Approving Institutional Review Boards**

● Beth Israel Deaconess Medical Center: ID 2010P-000412

● Boston Veterans Administration: ID 2540

● Harvard Medical School: ID M20493-101 (ceded to the Beth Israel Deaconess Medical Center through the Harvard Catalyst Regulatory Foundation, Ethics, and Law Program).

● Brigham and Women’s Hospital: ID 548 (ceded to the Beth Israel Deaconess Medical Center through the Harvard Catalyst Regulatory Foundation, Ethics, and Law Program).
