# Supplementary figures and images for: Tai chi mind-body exercise in patients with COPD: study protocol for a randomized controlled trial
Source: Trials. 2014 Aug 28;15:337. doi: 10.1186/1745-6215-15-337 (PMC4158042; doi:10.1186/1745-6215-15-337)

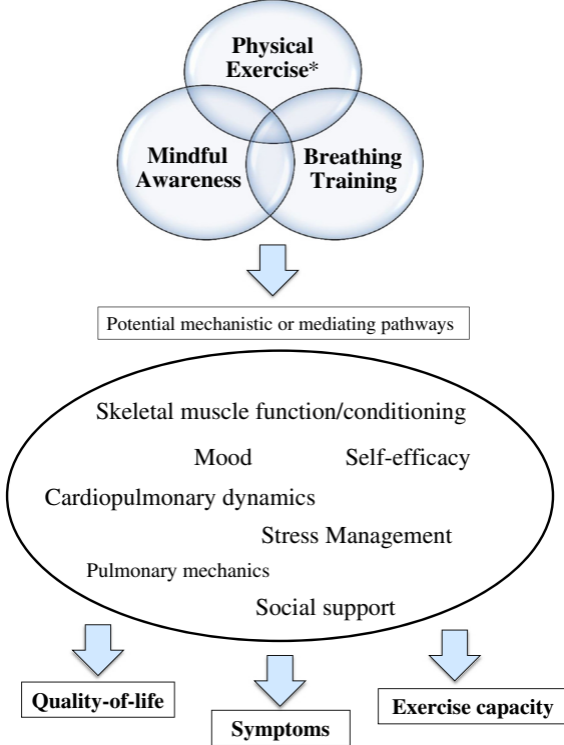

\*upper/lower extremity, core strengthening/stretching, dynamic structural integration

Supplement: Supplementary file 2 — Authors’ original file for figure 1 [file 13063_2014_2212_MOESM2_ESM.pdf]

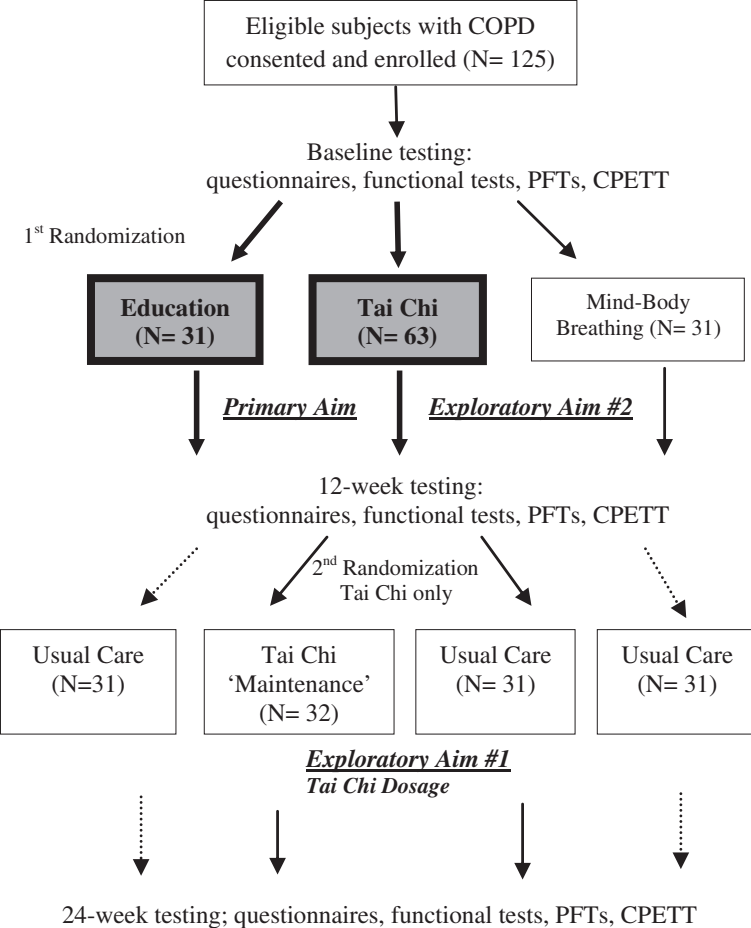

Supplement: Supplementary file 3 — Authors’ original file for figure 2 [file 13063_2014_2212_MOESM3_ESM.pdf]
